# Biochemical and Mass Spectrometric Characterization of Human *N*-Acylethanolamine-Hydrolyzing Acid Amidase Inhibition

**DOI:** 10.1371/journal.pone.0043877

**Published:** 2012-08-31

**Authors:** Jay M. West, Nikolai Zvonok, Kyle M. Whitten, Subramanian K. Vadivel, Anna L. Bowman, Alexandros Makriyannis

**Affiliations:** Northeastern University, Center for Drug Discovery, Boston, Massachussetts, United States of America; Griffith University, Australia

## Abstract

The mechanism of inactivation of human enzyme *N*-acylethanolamine-hydrolyzing acid amidase (hNAAA), with selected inhibitors identified in a novel fluorescent based assay developed for characterization of both reversible and irreversible inhibitors, was investigated kinetically and using matrix-assisted laser desorption/ionization time-of-flight mass spectrometry (MALDI-TOF MS). 1-Isothiocyanatopentadecane (AM9023) was found to be a potent, selective and reversible hNAAA inhibitor, while two others, 5-((biphenyl-4-yl)methyl)-*N,N*-dimethyl-2*H*-tetrazole-2-carboxamide (AM6701) and *N-*Benzyloxycarbonyl-L-serine β-lactone (*N-*Cbz-serine β-lactone), inhibited hNAAA in a covalent and irreversible manner. MS analysis of the hNAAA/covalent inhibitor complexes identified modification only of the N-terminal cysteine (Cys126) of the β-subunit, confirming a suggested mechanism of hNAAA inactivation by the β-lactone containing inhibitors. These experiments provide direct evidence of the key role of Cys126 in hNAAA inactivation by different classes of covalent inhibitors, confirming the essential role of cysteine for catalysis and inhibition in this cysteine N-terminal nucleophile hydrolase enzyme. They also provide a methodology for the rapid screening and characterization of large libraries of compounds as potential inhibitors of NAAA, and subsequent characterization or their mechanism through MALDI-TOF MS based bottom up-proteomics.

## Introduction

Current pharmacological strategies in drug development targeting the endocannabinoid system are focused on the discovery of therapeutic agents that selectively modulate the cannabinergic signaling for the treatment of human disorders, without being accompanied by undesirable psychotropic side effects [1], [2], [3]. Toward this goal, two principal endocannabinoid enzymes, fatty acid amide hydrolase (FAAH) and monoacylglycerol lipase (MGL), are promising candidates for drug discovery, by modulating the effects of the two principal endocannabinoids, anandamide (AEA) and 2-arachidonylglycerol (2-AG), respectively, at the cannabinoid receptors 1 and 2 (CB1 and CB2) [4], [5]. Other important bioactive fatty acid amides, such as *N*-palmitoylethanolamine (PEA), *N*-oleoylethanolamine (OEA) and oleamide (OA), with no or very low affinity for the cannabinoid receptors, are potential substrates in FAAH and *N*-acylethanolamine-hydrolyzing acid amidase (NAAA) regulated metabolism. NAAA is a lysosomal enzyme that carries out hydrolysis of fatty acid amides, with its highest activity against PEA [6]. It has been suggested that the anti-inflammatory, analgesic, and neuroprotective properties of PEA are mainly due to the activation of peroxisome proliferator-activated receptor alpha (PPAR-α), and also in part by activation of the GPR55 and GPR119 receptors [7], [8], [9]. Therefore interest in NAAA as a target for novel therapeutics has been increasing of late [10], [11], [12], yet unlike FAAH and MGL there is a complete lack of direct structural information on the enzyme. The amino acid sequence of hNAAA has 34% identity with human acid ceramidase (hAC), another lysosomal enzyme that is not well characterized, which hydrolyzes ceramide to sphingosine and free fatty acid [13], [14]. A very limited homology of hNAAA and hAC to conjugated bile acid hydrolase (CBAH) from *Clostridium Perfringens*, with an available X-ray structure [15], has been used to generate homology models for both proteins [12], [16]. All three enzymes belong to the cysteine N-terminal nucleophile (Ntn) hydrolase superfamily, each with the highly conserved amino acid catalytic triad of Cys, Arg and Asp [17], [18]. CBAH is a single chain protein whose biological assembly is a homotetramer [15], whereas hNAAA and hAC after self-catalyzed cleavage of the zymogen at the catalytic nucleophile Cys126 and Cys143, respectively, under acidic conditions form α- and β- subunits [16], [19]. The hNAAA mutant with Cys126 to serine substitution was resistant to self-proteolysis and remained inactive [19]. The precursor zymogen is most likely inactive but the active mature form of the enzyme is a heterodimer, consisting of a non-covalent complex of the α- and β-subunits [19], [20]. Site-directed mutagenesis identified four actual *N*-glycosylation sites in hNAAA, which were confirmed by our laboratory in a recent study using mass spectrometry [19], [20]. A putative hNAAA catalytic triad of amino acid residues Cys126, Arg142 and Asp145 has been predicted from the highly conserved nature of these residues in the cysteine Ntn hydrolase superfamily and site-directed mutagenesis experiments, which also identified Glu195 as a key determinant of acidic cleavage, and suggested that Asn287 plays an important role in proteolytic zymogen activation [19], [21]. Recently a mechanism of catalysis via a zwitterionic N-terminal cysteine in CBAH was proposed based on computational analyses of free energy simulations and suggested that NAAA may cleave its substrates using the same catalytic strategy [22].

Previously we overexpressed hNAAA in stably transfected HEK293 cells and purified the enzyme in an amount sufficient for its biochemical and proteomic characterization [20]. We have further optimized hNAAA expression to obtain a three-fold increase of hNAAA yield. Additionally we developed novel high throughput fluorescent inhibitor assays for characterization of both reversible and irreversible hNAAA inhibitors and identified several potent inhibitors in our compound libraries. 1-isothiocyanatopentadecane (AM9023) is a potent, selective and reversible hNAAA inhibitor, while 5-((biphenyl-4-yl)methyl)-*N,N*-dimethyl-2*H*-tetrazole-2-carboxamide (AM6701) and *N-*Benzyloxycarbonyl-L-serine β-lactone (*N-*Cbz-serine β-lactone) inhibit hNAAA in a covalent and time-dependent manner. The mechanisms of hNAAA inactivation by AM9023, AM6701 and *N-*Cbz-serine β-lactone were investigated using kinetic and MS experimental approaches. MALDI-TOF analysis of the tryptic digest of hNAAA treated with AM6701 or *N-*Cbz-serine β-lactone inhibitor identified modification only for the N-terminal cysteine (Cys126) of the β-subunit.

## Materials and Methods

### Materials

Standard laboratory chemicals, buffers, culture media and media components were purchased from Sigma-Aldrich and Fisher Chemical. AM6701 was made as previously described [23]. *N-*Cbz-serine β-lactone was purchased from TCI America.

### Synthesis of 1-isothiocyanatopentadecane, AM9023

1,1′-thiocarbonyldipyridin-2(1H)-one (65 mg, 0.28 mmol) was added to a suspension of 1-aminopentadecane (50 mg, 0.22 mmol) in 5 mL of anhydrous CH_2_Cl_2_. Upon completion (0.5 h) the reaction was quenched with water and the organic layer was separated and concentrated. The resulting residue was chromatographed on silica to yield AM9023 (55 mg, 94%) as a colorless oil. ^1^H NMR (500 MHz, CHLOROFORM-d) δ 3.51 (t, *J* = 6.80 Hz, 2H), 1.69 (td, *J* = 6.84, 15.14 Hz, 2H), 1.36–1.44 (m, 2H), 1.21–1.34 (m, 22H), 0.88 (t, *J* = 6.84 Hz, 2H). ^13^C NMR (126 MHz, CHLOROFORM-d) δ 127.3, 45.3, 32.2, 30.2, 30.0, 29.96, 29.94, 29.92 (2C), 29.8, 29.7, 29.6, 29.1, 26.8, 23.0, 14.4. IR (neat) cm^−1^ 2924, 2854, 2185, 2090. HRMS for C_16_H_30_NS (M-H^+^) 268.2111. Calcd. 268.2099.

### hNAAA overexpression and purification

The hNAAA expression and purification was performed as previously described, with the exception that ammonium chloride stimulated secretion of zymogen into media was repeated three times for the same cells, effectively tripling our hNAAA yield [20]. In brief, HEK293 cells stably expressing human NAAA with a C-terminal hexa-histidine tag were cultured at 37°C in a humidified incubator (5% CO_2_) on 500 cm^2^ plates in DMEM with 10% FBS, 1% penicillin-streptomycin (P/S), and 0.6 mg/mL Geneticin to approximately 90% confluency. The FBS containing culture medium was exchanged for 50 ml (per culture plate) serum-free DMEM with 1% P/S, 0.6 mg/mL Geneticin, 10 mM NH_4_Cl, and allowed to incubate for 48 hours. This step was repeated two more times at 48 hour intervals, with the medium centrifuged to remove cells and debris and the proteins were precipitated by adding ammonium sulfate to 60% saturation. The remainder of the purification was as previously described [20]. The day that assays or covalent labeling were performed 100 mM citrate-phosphate buffer, pH 4.5, was added to purified NAAA at a 4∶1 v/v ratio and incubated for 2 hours at 37°C in order to activate the enzyme.

### Fluorometric assay to determine hNAAA inhibition using N-(4-methyl coumarin)palmitamide (PAMCA) substrate

We previously described the fluorogenic substrate *N*-(4-methyl coumarin)palmitamide (PAMCA), which is hydrolyzed by NAAA to the fluorescent compound 7-amino-4-methyl coumarin (AMC) and palmitic acid [20]. For hNAAA inhibition we conducted three point concentration assays with compounds to determine their potencies and ranges of enzyme inhibition. Purified activated NAAA (final concentration of 0.25 µg/mL) was incubated in assay buffer [20] made up to a total volume of 180 µL, followed by addition of the compound dissolved in 10 µL DMSO (along with DMSO neat for the control sample) with the final concentrations for each compound of 1, 10, and 100 µM, in triplicate on a 96 well plate. These samples were allowed to incubate for 15 min at room temperature and then 10 µL of a PAMCA stock solution in DMSO (final PAMCA concentration 10 µM) was added. After 5 minutes of agitation on a shaking plate, the reaction was allowed to proceed at 37°C for 30 minutes and enzyme activity was monitored and calculated as previously described [20].

For compounds that inhibited hNAAA in the range of IC_50_<1 µM full inhibition curves using eight different concentrations of inhibitor (8 point assay) were generated. To set up 8 point fluorescent and radioactive assays for each point, the compound in 45 µL DMSO and purified activated NAAA (final enzyme concentration of 0.25 µg/mL) in 810 µL of NAAA assay buffer were incubated for 2 hours in order for the covalent compounds to reach full inhibition. For the fluorescent assays, 190 µL of each of the above samples (in triplicate) were transferred to a 96 well plate, followed by addition of 10 µL of a PAMCA stock solution in DMSO for a final PAMCA concentration of 10 µM. After 5 minutes of agitation on a shaking plate, the reaction was allowed to proceed at 37°C for 30 minutes and enzyme activity was monitored and calculated as previously described [20]. The complete description of the novel fluorescent 8 point assay to determine the IC_50_ value for compounds inhibiting hNAAA activity will be detailed elsewhere.

### Radioactive assay to determine *IC_50_* value for reversible NAAA inhibition using [^1,2 −14^C]*N*-palmitoylethanolamine substrate

The 8 point radioactive assays, similar to that described by Saturnino *et. al.*, [10] were performed by taking 95 µL of the enzyme-compound solution described above and adding a 5 µL solution of [^1,2 −14^C]*N*-palmitoylethanolamine (10,000 c.p.m./sample) in DMSO, with a final PEA concentration of 25 µM. The reaction was performed for 30 minutes at 37°C and terminated by addition of 200 µL chloroform/methanol (1∶1, v/v). The aqueous layer containing the [^1,2 −14^C]Ethanolamine was quantified by reading with a 1450 Microbeta Walluc Trilux Liquid Scintillation and Luminescence Counter. Inhibition constants were calculated using pro Fit software (Quantum Soft, Uetikon am See, Switzerland) and a Levenberg-Marquardt algorithm.

### Fluorometric assay to determine *k_inact_/K_i_* value for irreversible hNAAA inhibition

The *k*_inact_ and *K*_I_ values for covalent inhibitors were determined similar to Mileni *et. al*. [24] The fluorescence-based assays were performed as above with the following exceptions: fluorescence readings were initiated immediately (data were collected for 120 minutes at 30 second intervals) after mixing the inhibitor compound and PAMCA substrate (final concentration of 12.4 µM = 2×*K*_m_) with NAAA in the assay buffer using a PerkinElmer Wallac EnVision 2104 Multilabel reader to monitor the fluorescence. The data for each inhibitor concentration ([I]) were fit to a first order equation (Eq. 1) shown below using KaleidaGraph (Synergy Software, Reading, PA) in order to determine *k*_observed_ (*k*_obs_), where *F_t_* is the fluorescence at time *t*, *F*_0_ is the fluorescence at *t* = infinite time, *F*_1_ is the total fluorescence change, and *k*_obs_ is the first order rate constant for enzyme inactivation. To determine the inhibitor dissociation constant (*K*_I_) and the first order rate constant for enzyme inactivation at infinite inhibitor concentration (*k*_inact_), the *k*_obs_ values for each [I] obtained above were fit to a curve using pro Fit software and a Levenberg-Marquardt algorithm according to Eq. 2, which simplifies to Eq. 3 at [S] = 2×*K*_m_ as used in this experiment.
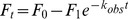
(1)

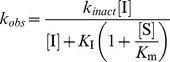
(2)

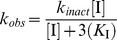
(3)


### Trypsin digestion of hNAAA treated with inhibitors

To 10 µg (0.2 nmol) of purified and activated NAAA in 18 µL of 100 mM citrate-sodium phosphate buffer (pH 4.5) 2 µL of a DMSO solution containing 2 nmol of the compound of interest were added or 2 µL DMSO. The inhibitor and DMSO treated enzyme solutions were incubated at 37°C for 2 hours and then desalted prior to digestion. These were desalted by re-concentrating 3 times to original volume after 25 fold dilution with 50 mM ammonium bicarbonate buffer, pH 8.0, using 10 kDa membrane Ultra-0.5 Centrifugal Filters (Millipore). The NAAA samples were incubated overnight at 37°C with MS-grade trypsin (“Trypsin Gold”, Promega) at a NAAA:trypsin mass to mass ratio of 100∶1. The tryptic digested NAAA was analyzed immediately or frozen at −80°C for future analysis.

### MALDI-TOF-MS Analysis

0.5 µL of the trypsin digested NAAA was mixed with 0.5 µL α-cyano-4-hydroxycinnaminic acid matrix solution (5 mg/mL dissolved in 50% acetonitrile, 50% water, and 0.1% trifluoroacetic acid) and spotted onto an Opti-TOF 384-well plate insert. MALDI-TOF MS spectra were acquired on a 4800 MALDI TOF/TOF mass spectrometer (Applied Biosystems, Foster City, CA) fitted with a 200-Hz solid state UV laser (wavelength 355 nm). The spectra of the peptides were acquired in reflectron mode. The conditions used for the MS experiments and instrument calibration were performed as described by Zvonok *et. al*. [25]

### Molecular Modeling

The sequence for human NAAA was taken from the SWISS-PROT protein sequence database (amino acids 126–359 primary accession number Q02083). The homology model of hNAAA was constructed using the crystal structure of conjugated bile acid hydrolase (CBAH) from *Clostridium Perfringens* (PDB ID: 2BJF) [15] as a template in Prime (1.6 ed., Schrödinger, LLC, New York, NY). An initial BLAST alignment between the two sequences was adjusted by taking secondary structure into account using SSpro and PSIPRED [26]. This alignment was further refined manually to superimpose Asn204 and Asn287 of hNAAA with Asn82 and Asn175 of CBAH respectively as previously suggested [12]. The resultant alignment (13% identity, 21% homology, 34% gaps) was used for construction of the initial hNAAA model. Loops 2–6 and 8–14 were refined using an *ab initio* loop prediction algorithm. The loop refinement step deletes the loop and reconstructs it from a backbone dihedral library; the loop is then exhaustively sampled to identify the lowest energy conformation. All other loops featured mainly homologous residues and contained no gaps or insertions. The protein underwent a truncated-Newton energy minimization, using the OPLS_2005 all-atom force field and a Generalized Born continuum solvation model.

AM6701 and *N*-Cbz-serine β-lactone were prepared for docking using the LipPrep (2.2 ed., Schrödinger, LLC, New York, NY) protocol and the OPLS_2005 force field. The ligands were docked to hNAAA using the extra precision (XP) procedure in Glide (5.6 ed., Schrödinger, LLC, New York, NY). The top pose for each ligand was then used to create the product for reaction. A covalent bond was imposed between the carbonyl carbon of the ligand and the sulfur atom of Cys126, for AM6701 the leaving group was removed and for N-Cbz-serine β-lactone the ring was opened. Atom types were reassigned and the entire system underwent minimization.

## Results and Discussion

### hNAAA overexpression and purification

The multiple harvesting of media containing the secreted enzyme, after the stimulation of lysosomal protein secretion via ammonium chloride treatment of the HEK293 cells, was used to increase the yield of overexpressed hNAAA. With this modification the stably transfected HEK293 cells with hNAAA construct produces ∼1 mg of IMAC purified enzyme per 5×10^3^ cm^2^ culture plate area (10×500 cm^2^ culture plates). This amount of enzyme is sufficient for 2×10^4^ data points in fluorescence-based inhibition assays (∼200×96 well plates) with 50 ng enzyme per well, enough to generate full inhibition curves for approximately 800 compounds. All other steps of hNAAA purification were similar to previously described.^16^

### Kinetic analysis of hNAAA inhibition by AM9023, AM6701, and *N-*Cbz-serine β-lactone

**Figure 1:**
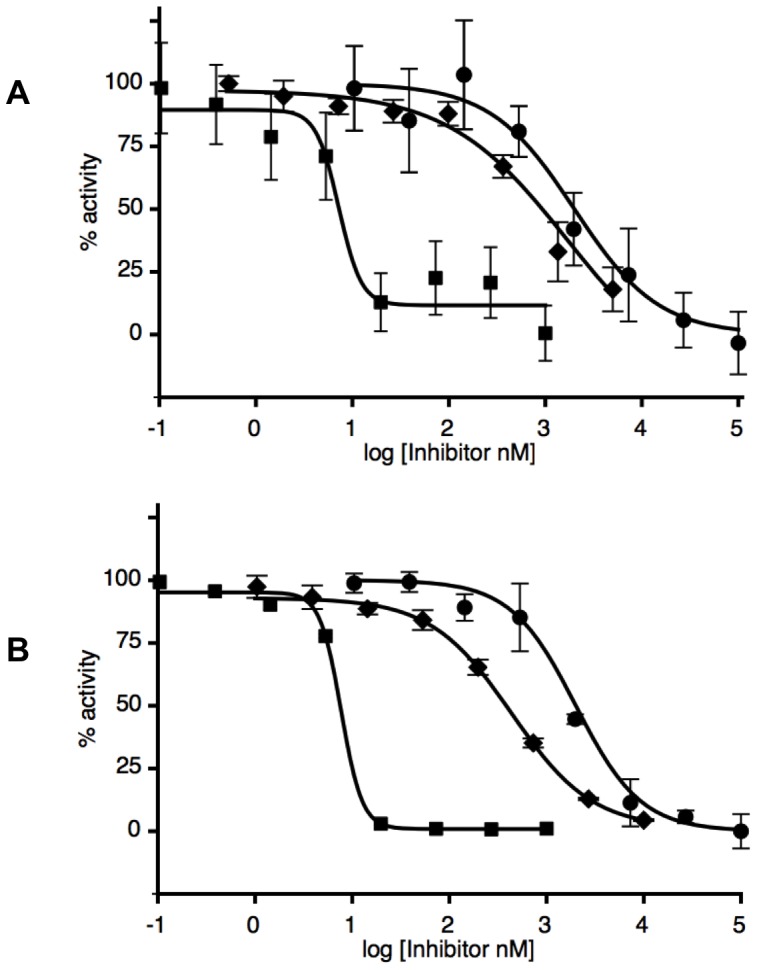
Concentration dependent inhibition of purified hNAAA by three compounds. hNAAA was incubated with the compounds AM6701 (squares), *N-*Cbz-serine β-lactone (circles), and AM9023 (diamonds) for two hours in order to reach full inhibition before measuring activity. Panel (A). A radioactivity-based assay with [^14^C] PEA as substrate. Panel (B). A fluorescence-based assay with PAMCA as substrate. Representative curves are displayed.

We previously introduced the novel fluorogenic compound *N*-(4-methyl coumarin) palmitamide (PAMCA), which has an affinity for hNAAA comparable to the native substrate PEA (*K_m_* 6.2 µM and 21 µM for PAMCA and PEA, respectively), and which is enzymatically hydrolyzed to the fluorescent 7-amino-4-methyl coumarin (AMC) and palmitic acid [20]. Although the rate of PAMCA versus PEA hydrolysis is two orders of magnitude slower the sensitivity, set up time, safety, and rapid readout of the fluorescence assay makes it superior to the radioactivity based assay methods. Therefore, PAMCA was selected as a substrate to develop a high throughput fluorescent inhibition assay to discover novel hNAAA inhibitors, similar to assays with FAAH and MGL enzymes [25], [27]. We first performed 3 point assay screens of our compound library to identify potential inhibitors of PAMCA hydrolysis by hNAAA. The enzyme and compounds at concentrations of 1, 10 and 100 µM (3 point assays) were pre-incubated for 15 min followed by addition of the substrate PAMCA and then monitoring the increase in fluorescence. For selected compounds we performed 8 point assays, shown in Figure 1, to obtain full inhibition curves and IC_50_ values. AM9023, AM6701 and *N-*Cbz-serine β-lactone, identified in the 3 point assay, were characterized both in radioactive (^14^C labeled PEA) and fluorescent 8 point assays to validate the observed IC_50_ values. The IC_50_ values for each selected inhibitor are very similar between those two assays and are presented in Table 1.

**Table 1:** Potencies of hNAAA inhibitors.

Inhibitor	IC_50_ [^14^C] PEA (nM)	IC_50_ PAMCA (nM)	*k_i_*_nact_ (min^−1^)	*K*_I_ (nM)	*k_i_*_nact_/*K*_I_ (M^−1^s^−1^)
AM6701	7.2±2.5	7.7±0.2	0.042±0.007	13±3	55000
*N*-Cbz-serine β-lactone	1900±500	1700±200	0.023±0.005	1300±400	290
AM9023	600±130	350±70	NA	NA	NA

The *k*_inact_ and *K*_I_ values for the covalent inhibitors were obtained as described in the Experimental Procedures. The IC_50_ values were calculated after 2 hours preincubation of the enzyme and inhibitor before addition of the substrate. Values are averages ± SD of three independent experiments.

### hNAAA inhibition by AM9023

**Figure 2:**
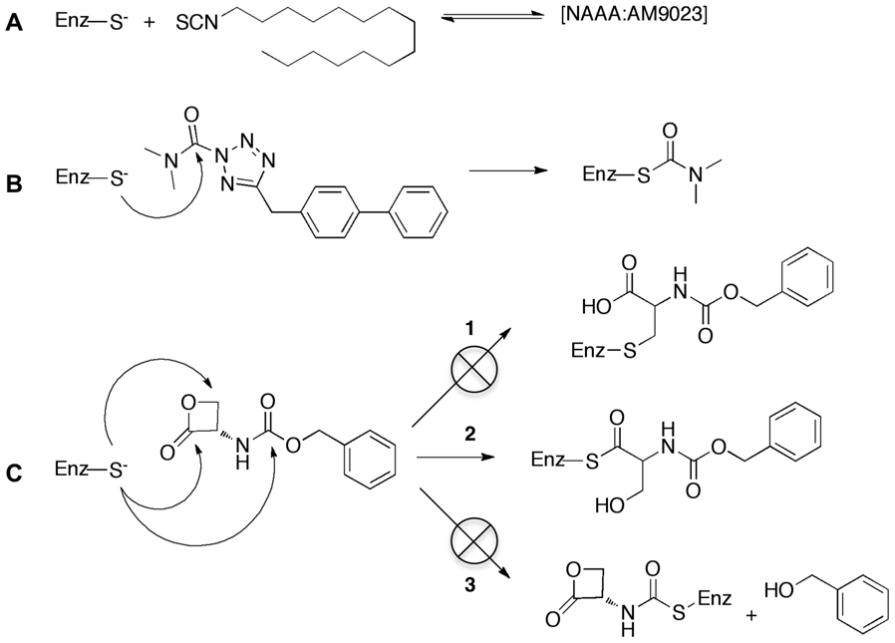
Putative mechanism of inhibition of hNAAA for three compounds studied. Panel (A). Reversible inhibition of hNAAA by AM9023. Panel (B). Irreversible inhibition of hNAAA by AM6701 via thiocarbamylation of Cys126. Panel (C). Irreversible inhibition of hNAAA by *N-*Cbz-serine β-lactone most likely proceeds via route 2.

AM9023 contains an isothiocyanate group (Figure 2a), and it was expected that it would react irreversibly with the cysteine nucleophile in the active site. We have used isothiocyanate based probes extensively to characterize the cannabinoid receptors, which covalently react with the cysteines in the receptor [28], [29], [30]. However, the following evidence suggested that AM9023 is a competitive reversible inhibitor: a) the IC_50_ was unaffected by longer pre-incubation of hNAAA with AM9023; b) enzyme activity was fully recovered in a rapid dilution experiment; c) the profile of a Lineweaver-Burk (double reciprocal) plot suggested it was a competitive type inhibitor. Yet AM9023 was a relatively potent inhibitor, with an IC_50_ = 350±70 nM as measured with the fluorescent assay and an IC_50_ = 600±130 nM obtained with the radioactivity based assay (Figures 1a and 1b and Table 1). AM9023 was selective for hNAAA as compared to hMGL or rFAAH, which each had an IC_50_>10 µM. These results suggested that AM9023 is a reversible and non-covalent inhibitor of NAAA, and mass spectrometric analysis was used to test this hypothesis in the mass spectrometric experimental section.

### hNAAA inhibition by AM6701

AM6701 was recently found to be a very potent inhibitor of both human MGL and FAAH, with both enzymes having equivalent IC_50_ values of 1.2 nM [31]. We identified AM6701 as also a very potent inhibitor of hNAAA with the IC_50_ values determined to be 7.2±2.5 nM (radioactive assay) and 7.7±0.2 nM (fluorescent assay) as shown in Figures 1a and 1b and Table 1. The AM6701 potency to inhibit hNAAA was time dependent; the longer enzyme was preincubated with inhibitor the lower IC_50_ was observed (extension of preincubation time to 120 min decreased the IC_50_ more than 50 fold; data not presented). A rapid dilution assay experiment with hNAAA and AM6701 was performed to determine if the inhibition was reversible. AM6701 was incubated with hNAAA at a concentration of 100 nM for 120 min, and after twenty-fold dilution to a final inhibitor concentration of 5 nM there was no recovery of enzyme activity, even after 24 h incubation, indicating that inhibition was irreversible, unlike the reversible inactivation of hMGL by AM6701 [23], [32].

### hNAAA inhibition by *N-*Cbz-serine β-lactone

Another compound, *N-*Cbz-serine β-lactone, initially discovered as a hepatitis A virus 3C proteinase inhibitor [33], and more recently identified as an inhibitor of rat NAAA with an IC_50_ in the low micro molar range, was characterized both in radioactive and fluorescent assays. Structure-activity relationship experiments suggested that the β-lactone portion of the compound was essential for inhibition [11], [12]. *N-*Cbz-serine β-lactone inhibited hydrolysis of the substrate heptadecenoylethanolamide by HEK293 cell lysate, containing overexpressed recombinant rat NAAA, with an IC_50_ = 2.96±0.3 µM as determined in LC/MS based assay [11], [12]. The IC_50_ inhibition values we obtained for purified hNAAA with the same inhibitor using the radioactive (IC_50_ = 1.9±0.5 µM) and fluorescent (IC_50_ = 1.7±0.2 µM) assays, as shown in Table 1, were similar. The potency of *N-*Cbz-serine β-lactone hNAAA inhibition was time dependent; however we observed a slow regeneration of enzyme catalytic activity in a rapid dilution experiment of hNAAA inhibited with this compound, similar to the previously reported partial recovery of initial enzyme activity (44±2%) following 12 h dialysis of rNAAA inhibited with *N-*Cbz-serine β-lactone analog.^12^ The recovery of enzyme activity after rapid dilution suggested that *N-*Cbz-serine β-lactone either introduces enzyme modification(s) that are reversible under assay conditions or it is a tight-binding reversible inhibitor. It was proposed that the β-lactone class of inhibitors inhibit NAAA via a covalent mechanism, that either alkylates or acylates the catalytic nucleophile cysteine via β-lactone ring opening (Figure 2c).

### Characterization of irreversible AM6701 and *N-*Cbz-serine β-lactone inhibitors of hNAAA

Variable pre-incubation time of the enzyme with the irreversible inhibitors AM6701 or *N-*Cbz-serine β-lactone resulted in a significant variation of IC_50_. To characterize this class of inhibitors the use of a second order rate constant, derived from the ratio of k_inact_/K_i_, has been suggested as the appropriate way to describe inhibitor potency [24], [34]. Unlike IC_50_ values, k_inact_/K_i_ are independent of pre-incubation times and therefore are a better measure of potency for irreversible inhibitors [24], [34]. To accurately determine k_inact_/K_i_ values it is necessary to follow the enzymatic reaction continuously, determining the concentration of either the substrate or product in the course of inhibition, which we can do with our fluorescence-based assay by monitoring the fluorescence at 460 nm, the emission peak of the product AMC. AM6701 was found to have a relatively high *k*_inact_/*K*_I_ of 55000 M^−1^ s^−1^ (Table 1), indicating that AM6701 is a very potent inhibitor. *N-*Cbz-serine β-lactone was much less potent with a *k*_inact_/*K*_I_ of 290 M^−1^ s^−1^. In summary, this novel fluorescent inhibition assay may be used for characterization of both reversible (based on IC_50_ values) and irreversible (based on *k*_inact_/*K*_I_ values) hNAAA inhibitors.

### MALDI-TOF MS analysis of hNAAA inhibition by AM9023, AM6701, and *N-*Cbz-serine β-lactone

To determine if the selected compounds covalently modified the enzyme, we employed LAPS methodology similar to that previously used with hMGL [23]. This approach consists of incubating the purified enzyme with and without a putative covalent inhibitor, evaluating extent of inactivation, performing a tryptic digest, comparing the peptide profile fingerprints using MALDI-TOF MS, and then assigning the site and nature of any covalent modification by MS/MS analysis.

### MALDI-TOF MS analysis of hNAAA inhibition by AM9023

The MALDI-TOF MS spectra of the tryptic digest of untreated and AM9023 treated hNAAA were identical (data not presented). This evidence along with the kinetic experiments strongly suggests that this isothiocyanate based compound is a reversible and non-covalent inhibitor of hNAAA.

### MALDI-TOF MS analysis of hNAAA inhibition by AM6701

**Figure 3:**
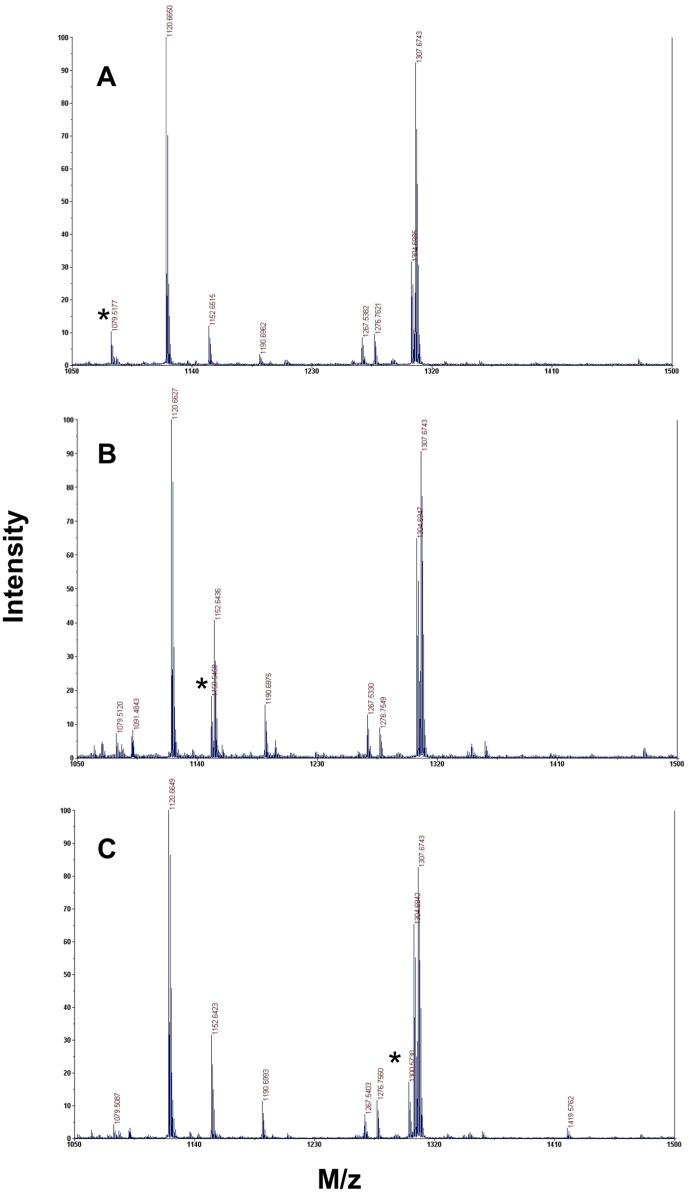
Tryptic digest fingerprint of purified hNAAA obtained by MALDI-TOF MS. Panel (A). Protein neat. Panel (B). AM6701 treated enzyme. Panel (C). *N-*Cbz-serine β-lactone treated enzyme. The T10-β peptide (sequence: CTSIVAQDSR, theoretical mass 1079.515 Da) peak containing the catalytic nucleophile cysteine and its covalently modified forms are marked with an asterisk in each panel.

**Figure 4:**
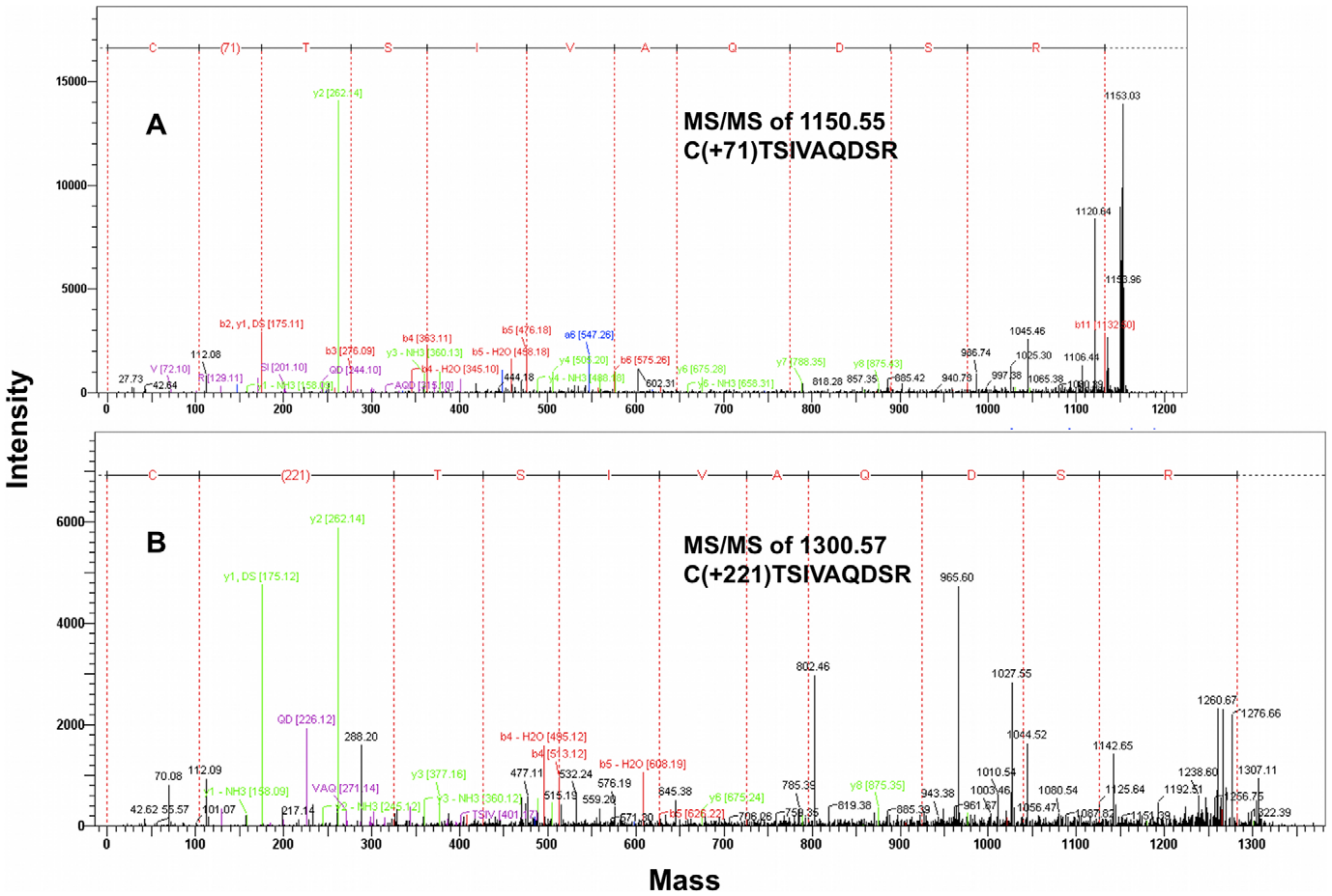
MALDI-TOF MS/MS analysis of the hNAAA tryptic peptide T10-β after covalent modification. Tandem MALDI-TOF MS/MS spectra of the T10-β peptide (sequence: CTSIVAQDSR) demonstrates covalent modification of Cys126 by both AM6701 (Panel (A)) and *N-*Cbz-serine β-lactone (Panel (B)).

**Figure 5:**
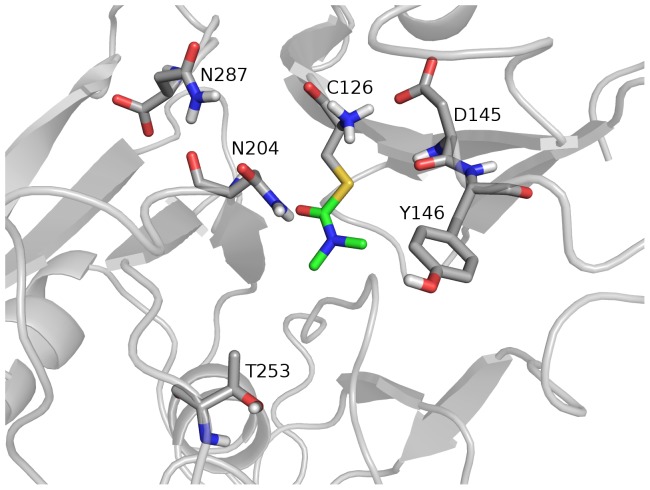
Representation of the active site of hNAAA after treatment with AM6701. Homology model illustrates thiocarbamylation of catalytic nucleophile Cys126 after treatment with AM6701.

MALDI-TOF MS analysis of the tryptic digests of untreated and AM6701 treated hNAAA identified a peptide with mass of 1079.5177 Da (T-10β peptide; CTSIVAQDSR), containing the catalytic cysteine in the control (untreated) sample, while a peptide with a mass of 1150.5459 Da was observed only in inhibitor treated samples (Figures 3a and 3b), with a concomitant decrease in the intensity of the unmodified T-10β peptide peak. The observed difference in mass, 71.0282 Da, between these two peptides is equivalent to the mass of a dimethylcarbamyl group (calculated 71.0371 Da) (Table 2). To confirm that the 1150.5459 Da ion was the T-10β peptide and determine which amino acid was covalently modified with a dimethylcarbamyl group, we performed an MS/MS analysis. The fragmentation data confirmed that this was the T-10β peptide (Figure 4a), and that the 71 Da additional mass was derived via carbamylation of the N-terminal cysteine. The putative covalent mechanism of AM6701 inhibition of hNAAA is shown in Figure 2b. To visualize the enzyme active site modified by AM6701, we constructed a hNAAA homology model based on the protein structure available in the protein data bank that it has the greatest homology in primary amino acid sequence with, which is with conjugated bile acid hydrolase (CBAH) from *Clostridium Perfringens*. Only the β-subunit was modeled with carbamylated catalytic nucleophile Cys126 because it contains the putative catalytic triad, and the α-subunit has no sequence homology with CBAH (Figure 5).

**Table 2:** Mass of tryptic peptide containing Cys126 of hNAAA after covalent modification.

Peptide sequence	Compound	*m/z* calculated	*m/z* measured	Error (ppm)
CTSIVAQDSR	DMSO (control)	1079.5150	1079.5177	2.5
CTSIVAQDSR	AM6701	1150.5521	1150.5459	−5.4
CTSIVAQDSR	*N*-Cbz-serine β-lactone	1300.5838	1300.5730	−8.3

T10-β peptides identified in the tryptic digest of untreated (control) and AM6701 or N-Cbz-serine β-lactone treated hNAAA samples.

### MALDI-TOF MS analysis of hNAAA inhibition by *N-*Cbz-serine β-lactone

**Figure 6:**
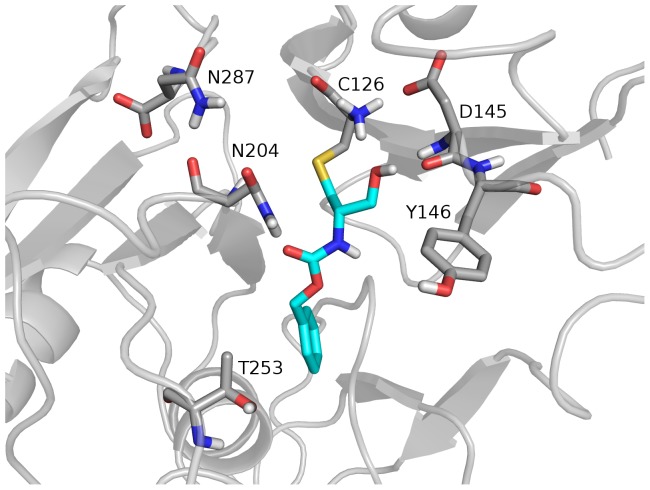
Representation of the active site of hNAAA after treatment with *N-*Cbz-serine β-lactone. Homology model illustrates acylated catalytic nucleophile Cys126 after treatment with *N-*Cbz-serine β-lactone.

Covalent modification by *N-*Cbz-serine β-lactone of the residue His102, in addition to the catalytic Cys172 in the active site, of hepatitis A virus 3C proteinase have been observed by NMR. Alkylation of Cys172 or N-alkylation of His102 occurred in hepatitis A virus 3C proteinase through the lactone cycle opening at the β-carbon (Figure 2c, route 1) [35]. The most likely mechanism of inhibition by *N-*Cbz-serine β-lactone with hNAAA is that the catalytic Cys126 in hNAAA is alkylated (route 1) or acylated (route 2) as shown in Figure 2c. A third possibility is Cys126 carbamylation, resulting in only a fragment of the compound covalently attached to the enzyme (Figure 2c, route 3). A slow regeneration activity of hNAAA inhibited with *N-*Cbz-serine β-lactone in a rapid dilution assay suggested acylation or carbamylation (route 2 and 3) rather than alkylation (route 1) of Cys126 (Figure 2c). Analysis of the MALDI-TOF MS spectra for the tryptic digests of untreated and *N-*Cbz-serine β-lactone treated hNAAA identified a difference in mass of 221.0553 Da between the peptides containing the catalytic cysteine in the control (untreated) sample (1079.5177 Da; T-10β peptide; CTSIVAQDSR) and inhibitor treated sample (1300.5730 Da), as shown in Figures 3a and 3c. The 1300.5730 Da ion was not observed in the untreated sample, and the unmodified T-10β peptide peak is still present but at a reduced intensity. The 221.0553 Da difference between these two peptides eliminated the possibility of route 3 in Figure 2c (calculated difference 131.0219 Da) and strongly suggested that the addition of *N-*Cbz-serine β-lactone (calculated 221.06888 Da, Table 2) must proceed by route 1 or 2 as shown in Figure 2c. The amino acid sequence of 1300.5730 Da ion identified by MS/MS analysis, as shown in Figure 4b, was confirmed to correspond to the T-10β peptide with Cys126 modified by the inhibitor. Our kinetic data demonstrating the low *in vitro* stability of *N-*Cbz-serine β-lactone treated hNAAA supports with the previous suggestion that a thioester bond is formed after attack of sulfur at the 2-carbonyl [11], as this is a more labile bond than the alkyl bond formed if the attack were at the 4-methylene, and hence is strong evidence that inhibition occurs by cysteine acylation via route 2 of Figure 2c. The homology model of hNAAA with the *N-*Cbz-serine β-lactone modified catalytic nucleophile Cys126, via acylation, is shown in Figure 6.

In the course of preparing this manuscript it was reported by Armirotti *et al*. that the β-lactones inhibit NAAA by S-acylation of the catalytic N-terminal cysteine [36], confirming our data presented in this manuscript and at the 2011 International Cannabinoid Research Society meeting [37].

## Conclusion

An understanding of structural organization and catalytic mechanism of the human enzyme N-acylethanolamine-hydrolyzing acid amidase is prerequisite to advance the development of medicines with anti-inflammatory, analgesic and neuroprotective properties. As the first step to hNAAA active site characterization we applied an MS-based ligand-assisted protein structure approach (LAPS) to identify an amino acid residue(s) in hNAAA susceptible to selected irreversible inhibitors. To obtain a sufficient amount of enzyme for the development, validation and executing of HTS inhibitor assays we further optimized a previously established HEK293-based hNAAA expression system to produce three-fold more secreted functional protein. Different classes of hNAAA inhibitors were pulled out during HTS screening of compound libraries using a 3 point fluorescence based assay, and the most potent were characterized further in a novel 8 point assay for reversible (based on IC_50_ values) and irreversible (based on *k*_inact_/*K*_I_ values) hNAAA inhibitors. The mechanisms of hNAAA inactivation by AM9023, AM6701 and *N-*Cbz-serine β-lactone were investigated in biochemical and MS experiments. The kinetics of hNAAA inhibition by AM9023 and MS analysis of untreated and AM9023 treated hNAAA strongly suggest that this isothiocyanate based compound is a reversible and non-covalent inhibitor of hNAAA. AM6701 and *N-*Cbz-serine β-lactone inhibit hNAAA in a covalent, time-dependent, and in the former case, irreversible manner. We observed slow partial activity recovery of hNAAA treated with *N-*Cbz-serine β-lactone, but not with AM6701 in a rapid dilution assay. MS analysis of untreated and AM6701 or *N-*Cbz-serine β-lactone inhibitor treated hNAAA samples, following trypsin digestion, identified modification only for the N-terminal cysteine (Cys126) of the β-subunit. These experiments confirm that hNAAA belongs to the cysteine N-terminal nucleophile class of enzymes, with Cys126 being the critical residue in the active site susceptible to covalent inhibitors, and establish methods to rapidly and efficiently determine the covalent or reversible nature of NAAA inhibitors and determine the potency of both types of inhibitors.

## References

[1] Ahn K, Johnson DS, Mileni M, Beidler D, Long JZ, et al (2009) Discovery and Characterization of a Highly Selective FAAH Inhibitor that Reduces Inflammatory Pain. Chemistry & Biology 16: 411–420. 19389627 10.1016/j.chembiol.2009.02.013 PMC2692831

[2] Alvarez-Jaimes LJ, Palmer JA The Role of Endocannabinoids in Pain Modulation and the Therapeutic Potential of Inhibiting their Enzymatic Degradation. Curr Pharm Biotechnol 10.2174/138920111798357357 21466449

[3] Saario SM, Laitinen JT (2007) Therapeutic potential of endocannabinoid-hydrolysing enzyme inhibitors. Basic & Clinical Pharmacology & Toxicology 101: 287–293. 17910610 10.1111/j.1742-7843.2007.00130.x

[4] Labar G, Michaux C (2007) Fatty acid amide hydrolase: From characterization to therapeutics. Chemistry & Biodiversity 4: 1882–1902. 17712824 10.1002/cbdv.200790157

[5] Saario SM, Laitinen JT (2007) Monoglyceride lipase as an enzyme hydrolyzing 2-arachidonoylglycerol. Chemistry & Biodiversity 4: 1903–1913. 17712832 10.1002/cbdv.200790158

[6] Tsuboi K, Sun YX, Okamoto Y, Araki N, Tonai T, et al (2005) Molecular characterization of N-acylethanolamine-hydrolyzing acid amidase, a novel member of the choloylglycine hydrolase family with structural and functional similarity to acid ceramidase. Journal of Biological Chemistry 280: 11082–11092. 15655246 10.1074/jbc.M413473200

[7] Godlewski G, Offertaler L, Wagner JA, Kunos G (2009) Receptors for acylethanolamides-GPR55 and GPR119. Prostaglandins Other Lipid Mediat 89: 105–111. 19615459 10.1016/j.prostaglandins.2009.07.001 PMC2751869

[8] Lo Verme J, Fu J, Astarita G, La Rana G, Russo R, et al (2005) The nuclear receptor peroxisome proliferator-activated receptor-alpha mediates the anti-inflammatory actions of palmitoylethanolamide. Molecular Pharmacology 67: 15–19. 15465922 10.1124/mol.104.006353

[9] O'Sullivan SE (2007) Cannabinoids go nuclear: evidence for activation of peroxisome proliferator-activated receptors. British Journal of Pharmacology 152: 576–582. 17704824 10.1038/sj.bjp.0707423 PMC2190029

[10] Saturnino C, Petrosino S, Ligresti A, Palladino C, De Martino G, et al (2010) Synthesis and biological evaluation of new potential inhibitors of N-acylethanolamine hydrolyzing acid amidase. Bioorganic & Medicinal Chemistry Letters 20: 1210–1213. 20022504 10.1016/j.bmcl.2009.11.134

[11] Solorzano C, Antonietti F, Duranti A, Tontini A, Rivara S, et al (2010) Synthesis and structure-activity relationships of N-(2-oxo-3-oxetanyl)amides as N-acylethanolamine-hydrolyzing acid amidase inhibitors. Journal of Medicinal Chemistry 53: 5770–5781. 20604568 10.1021/jm100582w PMC2932887

[12] Solorzano C, Zhu C, Battista N, Astarita G, Lodola A, et al (2009) Selective N-acylethanolamine-hydrolyzing acid amidase inhibition reveals a key role for endogenous palmitoylethanolamide in inflammation. Proc Natl Acad Sci U S A 106: 20966–20971. 19926854 10.1073/pnas.0907417106 PMC2791595

[13] Bernardo K, Hurwitz R, Zenk T, Desnick RJ, Ferlinz K, et al (1995) Purification, characterization, and biosynthesis of human acid ceramidase. J Biol Chem 270: 11098–11102. 7744740 10.1074/jbc.270.19.11098

[14] Tsuboi K, Takezaki N, Ueda N (2007) The N-acylethanolamine-hydrolyzing acid amidase (NAAA). Chemistry & Biodiversity 4: 1914–1925. 17712833 10.1002/cbdv.200790159

[15] Rossocha M, Schultz-Heienbrok R, von Moeller H, Coleman JP, Saenger W (2005) Conjugated bile acid hydrolase is a tetrameric N-terminal thiol hydrolase with specific recognition of its cholyl but not of its tauryl product. Biochemistry 44: 5739–5748. 15823032 10.1021/bi0473206

[16] Shtraizent N, Eliyahu E, Park JH, He X, Shalgi R, et al (2008) Autoproteolytic cleavage and activation of human acid ceramidase. Journal of Biological Chemistry 283: 11253–11259. 18281275 10.1074/jbc.M709166200 PMC2431059

[17] Brannigan JA, Dodson G, Duggleby HJ, Moody PC, Smith JL, et al (1995) A protein catalytic framework with an N-terminal nucleophile is capable of self-activation. Nature 378: 416–419. 7477383 10.1038/378416a0

[18] Oinonen C, Rouvinen J (2000) Structural comparison of Ntn-hydrolases. Protein Sci 9: 2329–2337. 11206054 10.1110/ps.9.12.2329 PMC2144523

[19] Zhao LY, Tsuboi K, Okamoto Y, Nagahata S, Ueda N (2007) Proteolytic activation and glycosylation of N-acylethanolamine-hydrolyzing acid amidase, a lysosomal enzyme involved in the endocannabinoid metabolism. Biochimica et Biophysica Acta 1771: 1397–1405. 17980170 10.1016/j.bbalip.2007.10.002

[20] West JM, Zvonok N, Whitten KM, Wood JT, Makriyannis A (2012) Mass Spectrometric Characterization of Human N-Acylethanolamine-hydrolyzing Acid Amidase. J Proteome Res 11: 972–981. 22040171 10.1021/pr200735a PMC3706083

[21] Wang J, Zhao LY, Uyama T, Tsuboi K, Tonai T, et al (2008) Amino acid residues crucial in pH regulation and proteolytic activation of N-acylethanolamine-hydrolyzing acid amidase. Biochimica et Biophysica Acta 1781: 710–717. 18793752 10.1016/j.bbalip.2008.08.004

[22] Lodola A, Branduardi D, De Vivo M, Capoferri L, Mor M, et al A catalytic mechanism for cysteine N-terminal nucleophile hydrolases, as revealed by free energy simulations. PLoS One 7: e32397. 10.1371/journal.pone.0032397 PMC3289653 22389698

[23] Zvonok N, Williams J, Johnston M, Panarinathan L, Karageorgos I, et al (2008) Covalent inhibitors of human monoacylglycerol lipase: Ligand-assisted proteomic characterization of the catalytic site. Chemistry and Biology 15: 854–862. 18721756 10.1016/j.chembiol.2008.06.008 PMC3972761

[24] Mileni M, Johnson DS, Wang ZG, Everdeen DS, Liimatta M, et al (2008) Structure-guided inhibitor design for human FAAH by interspecies active site conversion. Proceedings of the National Academy of Sciences of the United States of America 105: 12820–12824. 18753625 10.1073/pnas.0806121105 PMC2529035

[25] Zvonok N, Williams J, Johnston M, Pandarinathan L, Janero DR, et al (2008) Full mass spectrometric characterization of human monoacylglycerol lipase generated by large-scale expression and single-step purification. Journal of Proteome Research 7: 2158–2164. 18452279 10.1021/pr700839z PMC3689545

[26] Jones DT (1999) Protein secondary structure prediction based on position-specific scoring matrices. Journal of Molecular Biology 292: 195–202. 10493868 10.1006/jmbi.1999.3091

[27] Ramarao MK, Murphy EA, Shen MW, Wang Y, Bushell KN, et al (2005) A fluorescence-based assay for fatty acid amide hydrolase compatible with high-throughput screening. Anal Biochem 343: 143–151. 16018870 10.1016/j.ab.2005.04.032

[28] Li C, Xu W, Vadivel SK, Fan P, Makriyannis A (2005) High affinity electrophilic and photoactivatable covalent endocannabinoid probes for the CB1 receptor. J Med Chem 48: 6423–6429. 16190768 10.1021/jm050272i

[29] Morse KL, Fournier DJ, Li X, Grzybowska J, Makriyannis A (1995) A novel electrophilic high affinity irreversible probe for the cannabinoid receptor. Life Sci 56: 1957–1962. 7776819 10.1016/0024-3205(95)00176-7

[30] Picone RP, Khanolkar AD, Xu W, Ayotte LA, Thakur GA, et al (2005) (−)-7′-Isothiocyanato-11-hydroxy-1′,1′-dimethylheptylhexahydrocannabinol (AM841), a high-affinity electrophilic ligand, interacts covalently with a cysteine in helix six and activates the CB1 cannabinoid receptor. Mol Pharmacol 68: 1623–1635. 16157695 10.1124/mol.105.014407

[31] Naidoo V, Karanian DA, Vadivel SK, Locklear JR, Wood JT, et al (2012) Equipotent Inhibition of Fatty Acid Amide Hydrolase and Monoacylglycerol Lipase - Dual Targets of the Endocannabinoid System to Protect against Seizure Pathology. Neurotherapeutics 10.1007/s13311-011-0100-y PMC3480564 22270809

[32] Karageorgos I, Tyukhtenko S, Zvonok N, Janero DR, Sallum C, et al (2010) Identification by nuclear magnetic resonance spectroscopy of an active-site hydrogen-bond network in human monoacylglycerol lipase (hMGL): implications for hMGL dynamics, pharmacological inhibition, and catalytic mechanism. Molecular Biosystems 6: 1381–1388. 20464001 10.1039/c004515b PMC3697746

[33] Lall MS, Karvellas C, Vederas JC (1999) Beta-lactones as a new class of cysteine proteinase inhibitors: inhibition of hepatitis A virus 3C proteinase by N-Cbz-serine beta-lactone. Org Lett 1: 803–806. 10823207 10.1021/ol990148r

[34] Kitz R, Wilson IB (1962) Esters of methanesulfonic acid as irreversible inhibitors of acetylcholinesterase. J Biol Chem 237: 3245–3249. 14033211

[35] Yin J, Bergmann EM, Cherney MM, Lall MS, Jain RP, et al (2005) Dual modes of modification of hepatitis A virus 3C protease by a serine-derived beta-lactone: selective crystallization and formation of a functional catalytic triad in the active site. Journal of Molecular Biology 354: 854–871. 16288920 10.1016/j.jmb.2005.09.074 PMC7118759

[36] Armirotti A, Romeo E, Ponzano S, Mengatto L, Dionisi M, et al (2012) B-Lactones Inhibit N-acylethanolamine Acid Amidase by S-Acylation of the Catalytic N-Terminal Cysteine. ACS Medicinal Chemistry Letters 10.1021/ml300056y PMC4025845 24900487

[37] West JM, Whitten KM, Vadiei SK, Zvonok N, Makriyannis A (2011) Mass Spectrometric Characterization of Human NAAA. Research Triangle Park, NC, USA. International Cannabinoid Research Society P3–34.

